# A Suspected Case of Mixed Infection of *Plasmodium vivax* with *Plasmodium falciparum*: A Diagnostic Conundrum due to Pre-Analytical Error

**DOI:** 10.4269/ajtmh.20-0870

**Published:** 2020-11

**Authors:** Shreyam Acharya, Aparna Ningombam, Abhirup Sarkar

**Affiliations:** Department of Laboratory Medicine, All India Institute of Medical Sciences, New Delhi, India

India is highly endemic for malaria. Dual infections are reported infrequently. Peripheral smear microscopy is the gold standard of diagnosis to visualize the human cycle forms of *Plasmodium* (asexual forms and gametocyte). The sexual forms (ookinete and retorts) are usually seen in mosquitoes. Sexual forms and exflagellated forms of *Plasmodium vivax* are rarely seen in human blood. Here, we report an interesting finding of the ookinete form of *P. vivax* in human blood and how it may create morphological confusion with a gametocyte of *Plasmodium falciparum*.

A 5-year-old boy was admitted to the emergency ward with fever, chills, and rigor for 2 days. A lateral flow immunochromatographic test showed positivity against the pan-lactate dehydrogenase enzyme.

**Figure 1.**
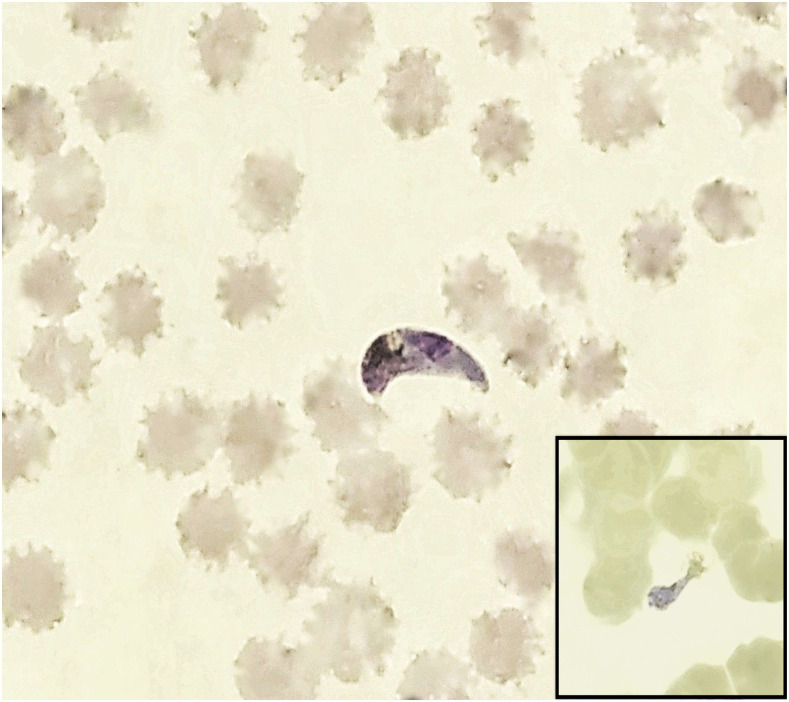
*Plasmodium vivax* ookinete with background RBCs showing storage changes in a thin smear. A retort form is also present (inset) (magnification ×1,000). This figure appears in color at www.ajtmh.org.

Giemsa-stained peripheral blood smears were made to confirm the diagnosis. Microscopy of the peripheral blood smear showed asexual forms, gametocyte of *P. vivax* with scattered crescent forms, creating a suspicion of dual infection with *P. falciparum*. Other intermediate forms such as exflagellated microgametocyte and zygote of *P. vivax* were also visible (Figure 1, inset).

Unlike *P. falciparum* gametocyte, crescents showed apical pigments, central nucleus, and vacuole, which is typical of ookinete of *P. vivax* (Figure 1).^1^ Because the initial slide preparation had a 6-hour delay from sample collection, blood samples were recollected and smears prepared immediately, which revealed the absence of ookinetes. Three examiners independently reviewed the slide and excluded the presence of *P. falciparum* of any stage.

To the best of our knowledge, the presence of ookinete in human peripheral blood has been reported once.^2^ Published literatures documenting the exflagellated microgametocyte mimicking *Borrelia* or other Spirochaetes and thereby creating a diagnostic perplexity have also been reported.^3–5^

Blood sample transport and slide preparation should not be delayed in suspected cases of malaria. The temperature of the laboratory also plays a significant role in the development of exflagellation and ookinetes. In our laboratory, the temperature was maintained at 24–25°C. Hence, this case highlights the importance of pre-analytical variables such as the time of collection and peripheral blood smear preparation in totality for the correct interpretation of peripheral blood smear findings. And last, this case reiterates the various morphological changes which may happen inside the vacutainer itself and how understanding the scenarios can help avoid a probable morphological confusion to the gametocyte of *P. falciparum* which can transpire.
